# Personality Traits, Technology-Related Teaching Skills, and Coping Mechanisms as Antecedents of Teachers’ Job-Related Affective Well-Being and Burnout in Compulsory and Higher Education Online Teaching Settings

**DOI:** 10.3389/fpsyg.2022.792642

**Published:** 2022-04-18

**Authors:** Rosana Stan

**Affiliations:** Department of Psychology, University of Oradea, Oradea, Romania

**Keywords:** online teaching, well-being, personality traits, digital competence, coping strategies

## Abstract

Teachers’ job-related well-being has been affected by the sudden shift to emergency remote online teaching due to the COVID-19 pandemic which has totally reshaped the task performance. Therefore, this study attempts to enlighten the possible reasons for the deterioration in teachers’ job-related well-being and proposes an integrated application of three models of prediction for job-related affective well-being and burnout as teachers’ indicators for the well-being in online teaching settings. The first model includes personality traits (extroversion, neuroticism, and conscientiousness) measured with the revised neuroticism, extroversion, and openness personality inventory (NEO-PI-R). The second model integrates an indispensable skill for the online teaching which is technological pedagogical content knowledge (TPCK) as technology-related teaching skill conceptualized by the TPACK framework. The TPACK model is a technology integration that identifies three types of knowledge instructors need to combine for successful EdTech integration - technological, pedagogical, and content knowledge (i.e., TPACK). The third model, a multidimensional one, includes coping mechanisms (e.g., problem-focused coping, emotion-focused coping, social support coping, and avoidant coping) as mediators in the relationship between personality traits and TPCK on the one side, and job-related well-being indicators on the other side. Findings from regression analyses were used to test the first two models, and the findings from a mediation analysis were used to test the third model to show that teachers’ TPCK explains a significant amount of variance in the job-related affective well-being of the teachers. The analyses also demonstrate that avoidant coping particularly mediates the relation between burnout and job-related affective well-being during COVID-19 school closures. Results indicate the efficacy of the TPACK model in increasing the job-related well-being of the teachers. The analysis of the data led to recommend that teachers should improve their personal technology-related teaching skills and adopt coping strategies in consistent with their personality traits. Moreover, public schools, as organizations, could advance educational technology programs to enhance technology-related teaching skills with the aim of increasing the well-being of their employees in online teaching settings.

## Introduction

Holmes et al. (2020) indicated that the most crucial consequence of remote working during the COVID-19 crisis is the work-related well-being of the employees. The difficulties in organizing distance learning became a source of stress for many teachers (Palareti, 2020). However, a systematic review comprising studies carried out before the pandemic (January 2005–December 2019) found that teachers present high levels of anxiety or stress due to their use of educational technology in the classroom (Fernández-Batanero et al., 2021). Specifically, the absence of prior training in online teaching techniques (Çoklar et al., 2016) or the pressure to acquire technological skills or the changes in the teaching methods (Amarilla and Vargas, 2009; Jena, 2015) have been previously proven to increase the stress of the teachers. In the actual pandemic context, it can be asserted that these educational technology demands might have consequences in the decrease and burnout growth of the teachers’ job-related affective well-being. Yet, independent of the type of stress, stress itself may be perceived differently by each teacher depending on the level of technological knowledge/resources (König et al., 2020) or skills, such as the self-efficacy to cope better with new and unexpected situations of high stress (Rabaglietti et al., 2021). Teachers’ subjective well-being is a topic often addressed in research (Garland et al., 2020), but the digital transition in the education domain has necessitated the analysis of well-being in online teaching settings. Subjective well-being is a composite of life satisfaction, high levels of positive affect, and low levels of negative affect (Diener, 1984), where affective well-being constitutes the core aspect of subjective well-being at work (Warr, 2007; Diener et al., 2009). Also, well-being is described as a multidimensional concept, with burnout as a specific job-related construct of poor well-being (Sonnentag, 2015) measured in a previous study as a negative indicator of the teachers’ well-being (Lauermann and König, 2016). Following Renshaw et al. (2015) who stated that research on teachers’ subjective well-being has targeted teacher burnout while neglecting the positive dimension of teachers’ subjective well-being, the present study approaches the job-related affective well-being which is considered to be the most researched positive indicator of the well-being of the teacher (van Horn et al., 2004), and burnout as a negative indicator of teacher functioning during online work or the negative side of employee well-being (Mäkikangas et al., 2016). Thus, in the current study, job-related well-being as a subjective well-being dimension was measured through two indicators, job-related affective well-being as the positive marker and burnout as the negative marker. However, how far the impact of technologies and digital services might affect people’s mental, physical, social, and emotional health, depends on the individuals’ personal context, circumstances, and their capacity to deal with or take advantage of the technologies and digital services (JISC, 2019). The COVID-19 pandemic has been described as an exceptionally uncertain situation that reveals dispositional characteristics of individuals (Judge and Zapata, 2015), such as personality traits that influence how people experience and perceive the world (Leger et al., 2021), or their characteristic adaptations related to coping styles (Waaktaar and Torgersen, 2010; Zager Kocjan et al., 2021) which lead the people either to successfully overcome the situation or make them unsuccessful in overcoming the situation. Thus, the individual’s response to a stressful situation is a complex result of the interactions among various factors where a psychological profile plays a key role (Steel et al., 2008) but cultural features are important as well (Biggs et al., 2017). Empirical arguments for the predictive role of personality traits for well-being was systematically analyzed (DeNeve and Cooper, 1998; Røysamb et al., 2008; Weiss et al., 2008; Anglim et al., 2020; Zager Kocjan et al., 2021) and determined that personality explained two-thirds of the variance in the affective dimensions of well-being (Josefsson et al., 2011). Consequently, there is a need for integrating the contributions of personality to well-being on the currently proposed models of teachers’ job-related well-being to establish the predictive role of other personal skills or characteristics over and above the personality traits. In addition, the COVID-19 pandemic is an exceptional situation regarding the switching of professional life to an online environment for many workers. Thus, not only knowledge but also technology-related teaching skills are required to use digital technologies efficiently during online teaching (Simons et al., 2017). As a result, digital skills have become essential for the teachers during this pandemic, but in most cases, teachers have not been trained in the necessary technical and pedagogical skills to integrate digital technology instruction (Schleicher, 2020). Therefore, the literature shows that implementing new technologies fundamentally affects individuals (Colbert et al., 2016) by forcing them to adopt the most efficient digital communication tools and to develop task-related digital competencies (Ter Hoeven et al., 2016). Solid evidence for how individual information communication technology (ICT) usage influences the employees’ well-being through shaping job demands, job autonomy, and relational aspects was identified (Wang et al., 2020). However, the effect of this exertion on the employees’ well-being depends on a series of factors. One previous study carried out in three Norwegian universities reveals that technology acceptance significantly impacts the academic employees’ work engagement as a dimension of work well-being (Shamsi et al., 2021). In the current pandemic, the Romanian education institutions and their employees shifted to remote work which involved new job demands, entailing a massive use of technologies throughout different types of videoconferencing activities like online teaching and an impressive reshaping of educational approaches (Nania et al., 2021). Moreover, Romania does not have any system with a long history of remote learning like other countries (i.e., Australia) (Dabrowski, 2020). For this reason, the Romanian educational staff may represent a “reference population” to investigate technology-related factors for job-related well-being. Although empirical evidence has shown that learning-induced demands can negatively affect the employees’ well-being (Wang et al., 2020), these detrimental effects can be alleviated in individuals with higher levels of technology self-efficacy (Tarafdar et al., 2015). Based on theoretical arguments presented above, the present study aimed to explore the role of an understudied technology-related factor, TPACK, on job-related well-being because, according to Mishra and Koehler (2006), knowledge related to digital technology and teaching content have been shown to be necessary for teachers when teaching with technology. In addition, a previous study argued that personality traits explain individual differences in stress reactions (Zager Kocjan et al., 2021) and that teachers’ coping responses to stressors were related to their well-being (Talbot and Mercer, 2019; Herman et al., 2020; MacIntyre et al., 2020). Although a considerable amount of research on well-being and coping strategies has been published, no one has studied the relationship between them in case of job-related well-being in online teaching settings. Based on the transactional model of stress (Lazarus and Folkman, 1984), one may also expect a moderating effect of coping strategies in the relationship between personality traits and knowledge related to digital technology in online teaching on one side and job-related well-being on the other side.

Since teachers do not know how long the pandemic will increase the amount of time they are required to spend online or what is in store for them in the future regarding the social distancing practices, the present study contributes to a better understanding of the mechanisms that affect teachers’ job-related well-being in online teaching settings. Specifically, the purpose of the current study is to assess the predictive role of the teachers’ technology-related teaching skills over and above personality traits and the function of coping mechanisms as mediators in the relationship between personality dimensions and the teachers’ job-related well-being during the COVID-19 pandemic. So far, the teachers’ technology-related teaching skills as the predictor and the multidimensional model of prediction including coping strategies as mediators for teachers’ job-related well-being indicators have not been studied in any previous studies. Consequently, the present study may interest the educators because it expands the research regarding the relationship between personality traits and teachers’ job-related affective well-being and explains the dynamic of the relationship between personality and the coping strategies of the teachers’ job-related well-being under conditions of intense or enduring stress, during the pandemic period. From a practical point of view, the improvement of teachers’ job-related well-being through psychological variables, such as coping strategies or technology-related teaching skills that can be learned, would enable positive outcomes related to well-being like long-time retention or high job performance. From a theoretical point of view, the present study aims to evaluate the risks or the potential benefits of increasing job-related affective well-being throughout the improvement of technological skills, even when the pandemic ends. Thus, the current study seeks to close an existing scientific gap in the literature regarding the role of personality traits, coping strategies, and technology-related teaching skills for reducing teachers’ burnout and increase job-related affective well-being by aiming to assess two major questions: (1) How is teachers’ job-related well-being affected by their technological pedagogical content knowledge? and (2) What impact do teacher coping strategies have on the job-related well-being in relation to teacher personality traits and/or technological pedagogical content knowledge level?

The research questions were developed to better understand the well-being as a job outcome in specific conditions enforced by the COVID-19 pandemic for a specific professional group, teachers from the Romanian public schools and the universities, with the aim of highlighting possible ways to increase the well-being in online teaching settings and to lead the way for future comparison in different cultural context. To answer the research questions, the conceptual framework for assessing the target variables is presented further.

### Subjective Well-Being in Online Teaching Settings

Subjective well-being refers to the extent to which a person believes or feels that his life is going well, including both cognitive evaluations and affective feelings (Diener et al., 2018). The present study taps the affective dimension of well-being in the work context. Much of the historical research on the teachers’ subjective well-being has targeted the teacher stress and burnout (Renshaw et al., 2015); while other studies have explored the utility of the positive subjective well-being indicators, such as positive emotions (van Horn et al., 2004). The present study was guided by the Hedonistic tradition in a conceptualized well-being (Deci and Ryan, 2008), measuring the balance of positive and negative emotions (Alexandrova, 2015). Although it is possible for someone to be experiencing both the indicators at the same time (Deci and Ryan, 2008), the positive subjective well-being indicators have been empirically supported as being distinct from and in addition to the negative indicators (van Horn et al., 2004). Even though researchers have found that well-being is relatively stable despite short-term fluctuations in response to transient events (Anglim et al., 2020), teachers are faced with new normalcy brought by the COVID-19 pandemic. The fact that many teachers have been asked to support their students through online practices has opened new ideas in terms of teacher well-being. As a result, educators must take into consideration the term, “well-being in online teaching settings.” A systematic narrative review (Best et al., 2014) found a variety of results with regard to how the online world may influence well-being. Research has indicated that remote workers work more (Aborg et al., 2002) and suffered from a perceived increase in workload (Molino et al., 2020). Although several humans–computer interaction studies underline the link between stress and individual well-being (Garcia-Ceja et al., 2016), and ways that digital technologies can affect mental processes (Passey, 2021), it is worth accentuating that there could be wider consequences in implementing digital technologies in the teaching settings. This might result in an interference with different indicators of well-being due to the complicated nature in the relationship between internet usage and subjective well-being (Ong et al., 2021). In addition, research has indicated that cultural context plays a major role in subjective well-being (Diener et al., 2018). Employing Warr’s (2002, 2007) model in examining the work-related well-being, the present study measures the well-being in online teaching settings focusing on job-related affective well-being and the burnout of Romanian teachers during the 2.5 months of the COVID-19 pandemic period. Job-related affective well-being targets the positive and negative effects which are defined as positive and negative emotions and moods that a person feels (Diener and Suh, 1997); while burnout is a syndrome conceptualized as resulting from chronic workplace stress that has not been successfully managed (World Health Organization, 2019). The burnout is a severe consequence of prolonged exposure to stress at work (Kalimo et al., 2003), a state characterized by deactivation and displeasure (Mäkikangas et al., 2016) as well as a syndrome characterized by emotional exhaustion, depersonalization, and lacking personal accomplishment (Maslach and Leiter, 2008). Utilizing Maslach and Leiter’s (2008) dimensional model of burnout, the current study analyzes emotional exhaustion and depersonalization as negative indicators of teachers’ well-being because they are considered the core components of burnout (Bakker et al., 2006). Thus, if job-related affective well-being specifically taps the affective dimension of well-being, burnout measures have been provided in the research literature, as well, when assessing the negative side of the employee well-being (i.e., Rothmann, 2008; Mäkikangas et al., 2016; Vîrgă and Iliescu, 2017). On the other hand, studies that examined the association between well-being and burnout have concluded that a reduced state of well-being might reflect the presence of burnout (Pillay et al., 2005; Milfont et al., 2008).

### Personality Traits as Predictors for Subjective Well-Being

Before the COVID-19 pandemic, the predictive validity of dispositional personality traits was more often examined in the general population under everyday circumstances. A previous study has shown that personality traits described by the five-factor personality model are related to subjective well-being (Anglim et al., 2020; Røysamb et al., 2008; Weiss et al., 2008) and this relationship is stronger particularly when neuroticism, extroversion, and conscientiousness are analyzed (DeNeve and Cooper, 1998). Extroversion usually has the highest correlations with the measures of well-being, while neuroticism typically has the highest correlations with the negative indicators of psychological functioning as a well-being indicator (Anglim et al., 2020). However, personality traits may be good candidates for explaining individual differences in stress reactions, including subjective well-being (Zager Kocjan et al., 2021). As a result, the personality traits were extensively analyzed recently as a predictor for changes in the perceived stress of the COVID-19 pandemic (i.e., Al-Omiri et al., 2021; Shokrkon and Nicoladis, 2021; Zacher and Rudolph, 2021). A recent study found that during the COVID-19 pandemic, individuals with high neuroticism worried more about the consequences of the pandemic and experienced more negative effects during this preoccupation (Kroencke et al., 2020). The present study follows this direction and analyzes the direct and indirect effects of personality traits (extroversion, neuroticism, and conscientiousness) for the well-being in online teaching settings. Considering that personality is critical to the experience of well-being (Anglim et al., 2020) and having unique physical distancing measures introduced during COVID-19 like online teaching, the following hypothesis was formulated:

*Hypothesis 1.* Extroversion and conscientiousness are positively correlated with job-related affective well-being; neuroticism is negatively correlated with job-related affective well-being (H1a); also, extroversion and conscientiousness are negatively correlated with burnout, while neuroticism is positively correlated with burnout (H1b).

### Technological Pedagogical Content Knowledge as Essential Dimension for Teacher Well-Being in Online Teaching Settings

Concerns about the appropriate uses of technologies in the teaching practices as well as the digital well-being that includes teachers have been studied in-depth (Diefenbach, 2018), but the link between the uses of digital technologies and how they might positively support the teacher well-being has received limited research attention (Passey, 2021). A previous study emphasizes that factors, such as the lack of technological knowledge (La Paglia et al., 2008; Wang and Li, 2019) or problems experienced with the use of technology (Al-Fudail and Mellar, 2008) contributed to an increased level of teachers’ stress. The pandemic has caused the need of integrating digital technologies into daily routines at an unprecedented rate (Dennis, 2021) and different types of knowledge related to digital technology, instruction, and teaching content are assumed to be necessary for teachers when teaching with technology (Mishra and Koehler, 2006). Technology-related teaching skills include identifying and using the appropriate technologies in a way that facilitates a broad range of learning activities relevant for students (Chi and Wylie, 2014). As many teachers had limited access to conventional teaching materials during the lockdown, those who had been trained in searching for and selecting online teaching materials may have better opportunities to provide support to their students. As a result, they might have been more confident and less stressed in online teaching settings. The literature already highlights that the lack of digital competencies among teachers has caused a high level of fatigue, stress, and a negative emotional state during the transition to online teaching (Oyedotun, 2020; Sokal et al., 2020; Mikuska, 2021). Studies have also shown that there is a significant variance in the skills of teaching professionals (Vähäsantanen and Hämäläinen, 2019) as well as a variation between and within countries to the degree to which the teachers use digital technologies in their work (Fraillon et al., 2019). Thus, technology-related teaching skills may count for an additional effect over and above personality traits in predicting burnout and job-related affective well-being.

The technological pedagogy content knowledge (TPACK) developed by Mishra and Koehler (2006) is the paramount framework in the present research when investigating the impact of technology-related teaching skills on teacher well-being in online teaching settings. The TPACK model of Mishra and Koehler extends Shulman’s (1986) perspective which postulates that teachers need a combined knowledge of content and pedagogy known as pedagogical content knowledge. Mishra and Koehler (2006) added a third component to Shulman’s (1986) model of pedagogical knowledge (PK) and content knowledge (CK) which is technological knowledge (TK). As a result, four hybrid components were formed at the intersections of the different knowledge areas, known as pedagogical content knowledge (PCK), technological pedagogical knowledge (TPK), technological content knowledge (TCK), and TPCK (Mishra and Koehler, 2006). Presently, the TPACK model is one of the most prominent models to enhance teacher knowledge for the effective use of digital technologies in teaching (Schmid et al., 2020). The central component of TPACK is TPCK because it is considered to arise from the integration of the other components of teacher knowledge. In this integrative view, high levels of TPCK will be constituted by high levels of TPK, TCK, PCK, TK, PK, and CK. On the other hand, in the transformative view, TPCK is considered more than the fusion of the core components and is regarded as a distinct form of knowledge. Irrespective of which approach is taken into consideration, if results remain inconclusive regarding the interplay of TPACK knowledge domains (Celik et al., 2014; Dong et al., 2015), the TPCK will provide a quicker and global assessment of knowledge required by teachers for integrating technology into their teaching in any content area. Consequently, from the survey on teachers of various subjects, a general perspective on teacher knowledge was measured using the TPACK model. At present, one of the most widely used self-report instruments is the survey developed by Schmidt et al. (2009) for assessing teachers’ TPACK knowledge domains. However, previous findings argue that the TPACK model influenced the existence of technostress in teachers and their disposition toward educational technology (Joo et al., 2016).

Therefore, teachers’ TPACK competence can be considered a decisive resource for teacher well-being during the adaptation to online teaching during COVID-19 school closures, and besides the traditional role of personality traits (extroversion, neuroticism, and conscientiousness), the technology-related teaching skills also play an essential role in employee well-being. Consequently, the following hypothesis was formulated:

*Hypothesis 2*. Technological pedagogical content knowledge is positively correlated with job-related affective well-being (H2a) and negatively correlated with burnout (H2b).

### The Role of Coping Strategies for Subjective Well-Being

Coping is the process of responding to a stressor using one or more available strategies for reducing, minimizing, or tolerating stress (Gustems-Carnicer and Calderón, 2013) and it comprises an effort to handle new situations that are likely to be demanding (Lazarus and Folkman, 1984; Lazarus, 2006) when limiting the concept of coping to voluntary responses (Compas et al., 2001). A rapid shift to online teaching, as a measure imposed during the COVID-19 pandemic, can be considered as being demanding, and it entails a deliberate effort to cope with a new type of teaching and networking with the students. Researchers have outlined that the individual differences in teachers’ coping skills are at the core of leading to teacher stress in addition to the competency in executing practices that effectively manage the teaching–learning process (Herman et al., 2020). Thus, teachers’ use of coping responses to stressors is an important determinant of their psychological adjustment and well-being (Talbot and Mercer, 2019).

Lazarus and Folkman (1984) divided the coping strategies into emotion-focused and problem-focused strategies in their “transactional model” of stress and behavioral self-regulation but Carver and Scheier (1998) argued that this distinction is too simplistic. Consequently, they developed a multidimensional model of coping accompanied by a measurement instrument, the coping orientation to problems experienced (COPE) Inventory (Carver et al., 1989). Carver et al. (1989) identified four dimensions/factors of coping: *coping focused on the problem* (including affective approach, planning and deletion of concurrent activities as coping strategies), *coping focused on emotions* (including positive interpretation and growth, abstention, and acceptance), *coping focused on search for social support* (covered by the use of social-instrumental support, social-emotional support, and focusing on expressing emotions as coping strategies) and *avoidance coping* (denial, mental, and behavioral deactivation as coping strategies). This study opted for the previously presented classification. Substance consumption, religious approach, and humor are coping strategies proposed by Carver et al. (1989) but they have not been included in the measured dimensions since these scales seemed to be rather heterogenous and independent ways of coping that are not related to a specific latent common factor on the Romanian population (Crasovan and Sava, 2013). Although Carver does not recommend viewing the COPE Inventory as a single scale to measure a general construct and emphasized that it is important not to preordain certain strategies as better than others (Carver et al., 1989), some researchers grouped coping strategies into larger constructs, such as “approach” and “avoidant” coping styles (Rosario et al., 2003). Approach strategies actively work to change the stressor or accept its presence in one’s life while avoidant coping strategies tend toward more dysfunctional responses, such as denial or distraction. Studies have shown that well-being correlates positively with the coping approach and negatively with avoidant coping (MacIntyre et al., 2020). A concern throughout the literature on stress and coping is how successful different coping strategies are in producing more positive outcomes, and lead to fewer negative outcomes. Research indicates that the option of a coping strategy is largely dependent on personal traits and although personality and coping are related, coping is not simply a direct manifestation of personality under adverse conditions (Carver and Connor-Smith, 2010). Other studies indicate that coping is less stable than personality and coping predicts adjustment over and above personality (Connor-Smith and Flachsbart, 2007), imposes the need to distinguish both concepts empirically, and to treat them independently, as different constructs. However, while the Big Five personality traits represent broad dispositional traits that describe a person’s behavior in many different contexts across time, coping mechanisms can be seen as a characteristic adaptation (Waaktaar and Torgersen, 2010). Self-regulation, for example, has been proposed as a mechanism for explaining the relationship between personality and subjective well-being (Carver and Scheier, 1990). Resilience is an underlying mechanism through which basic personality dimensions predict indicators of psychological functioning during the COVID-19 pandemic, including subjective well-being (Kocjan et al., 2021). Other studies also found a direct link between coping and burnout, where the role of coping is to alleviate the levels of emotional exhaustion and cynicism (Yip et al., 2008; González-Morales et al., 2010). Drawing on the arguments outlined above, it was investigated how successful different coping strategies were as mediators between personality traits and well-being indicators during online teaching in the COVID pandemic, and consequently, the following hypothesis was formulated:

*Hypothesis 3.* Problem-focused coping (H3a), emotion-focused coping (H3b), social support-focused coping (H3c), and avoidant coping (H3d) mediate the relationship between personality traits and teacher well-being indicators.

## Materials and Methods

### Sampling and Participants

The research was conducted on a convenience sample, and the responses were gathered using the snowball sample method, depending on teachers’ availability. Two hundred eighty-four teachers completed the online questionnaire. The mean age in the current sample is 43.37 (*SD* = 8.79). The detailed description of the sample from a socio-demographic perspective is presented in Table 1.

**TABLE 1:** The demographic profile of respondents (*N* = 284).

	Frequency	Percent
**Gender category**		
Male	44	15.5
Female	240	84.5
**Marital status**		
Single	74	26.1
Married/in a relationship	210	73.9
**Level of teaching**		
Pre-school and primary	81	28.5
Gymnasium and high school	128	45.1
University	75	26.4

### Procedure

Data were collected using Google Forms, a commonly used method during the pandemic (Di Monte et al., 2020; Maugeri et al., 2020). Since all responses were compulsory, no missing data were recorded. The survey was shared *via* social media networks of teachers and through personal email contacts between April 15 and June 30, 2021. The link containing the questionnaires was distributed to more than four hundred teachers working in pre-university and university education in Romania’s Western area. Before the first section of questions, the purpose of the study and ethical aspects relevant to the informed consent, before participating, was explained. Respondents were advised on the consent page not to take part or quit at any time if they felt uncomfortable thinking about their feelings or personal characteristics, and were informed about the necessary time for filling the survey (between 15 and 20 min). The main inclusion criteria were that all teachers have the legally required qualification for teaching in compulsory and higher education and to be full-time employed, according to the methodological norms in force. Since teaching with technology differs at baseline regarding educational stages, especially when comparing primary/elementary schools with universities (Fernández-Batanero et al., 2021), the existence of possible differences in the technological knowledge across the three categories of teachers were explored. No significant difference regarding TPCK was found between the teachers of primary, secondary, and tertiary levels of education. Thus, teachers from compulsory and university education were included together because it can be considered that they have the same background regarding adaptation to online teaching settings, irrespective of their teaching levels or subject areas of teaching. Moreover, all participants passed at the same time in online teaching setting without previous training in using Microsoft Teams, Moodle, or Zoom as ICT platforms adopted by the Romanian educational system. Further, the differences between all the levels of teaching in compulsory and university education regarding job-related affective well-being and burnout as job-related indicators were checked and no differences between the groups were found.

### Measures

Job-related affective well-being (JAW) was measured using the job-related affective well-being scale (JAWS) developed by Van Katwyk et al. (2000), which consisted of 10 positive and 10 negative job-related effects, the respondents had experienced in the last 30 days. The respondents were asked to choose one out of the five variable categories ranging from “never” to “extremely often.” Examples of the items include: “My job made me feel disgusted” or “My job made me feel inspired.” Higher scores on the scale indicate higher levels of job-related affective well-being. The scale was extensively used in other cultural contexts as a measure of job-related affective well-being (Schaufeli and van Rhenen, 2006; Basińska et al., 2014) and with Romanian employees as well (Cicei, 2012). It was checked for accuracy using the standard back-translation technique (Brislin, 1970). In terms of construct validity, confirmatory factor analysis (CFA) using R (R Core Team, 2021) revealed acceptable fit measures for the current sample [χ^2^ = 655.62, df = 169, *p* < 0.001; comparative fit index (CFI) = 0.89, Tucker–Lewis’s index (TLI) = 0.87, root mean square error of approximation (RMSEA) = 0.10 (0.09, 0.19), and standardized root mean square residual (SRMR) = 0.06]. Cronbach’s alpha value of the scale, as a reliability measure for the present sample, reveals an excellent internal consistency (α = 0.95).

Burnout was assessed with two scales of the Maslach Burnout Inventory-General Survey (Schaufeli et al., 1996), emotional exhaustion, and cynicism. Each scale is composed of five items. Examples of the items include: “I feel emotionally drained from my work” (exhaustion) and “I have become more cynical about whether my work contributes anything” (cynicism). All items were scored on a seven-point scale ranging from 0 (never) to 6 (always). The factorial validity of the Maslach Burnout Inventory—General Survey is similar across a wide variety of occupations of employees recruited through the Internet (Bakker et al., 2002). The instrument was previously validated on a Romanian population (Bria et al., 2014) and used as a measure for the negative dimension of job-related well-being not only in a Romanian sample (Vîrgă and Iliescu, 2017) but in different studies measuring the teachers’ well-being (Lauermann and König, 2016). The overall burnout score composed of exhaustion and cynicism scores was used in the study since it is considered that exhaustion and cynicism represent the core dimensions of burnout (Schaufeli and Taris, 2005). In terms of construct validity, CFA indicated very good fit measures [χ^2^ = 176.84, df = 34, *p* < 0.001, CFI = 0.92, TLI = 0.90; RMSEA = 0.12 (0.10, 0.14), SRMR = 0.05] for the present sample. Both the scales had excellent reliability measured through internal consistency (emotional exhaustion, α = 0.91; cynicism, α = 0.81) in the current sample.

Personality traits were assessed using the short version of the IPIP scales which measure three NEO-PI-R factors, such as extroversion, conscientiousness, and neuroticism in the short version. The scales were adapted for the Romanian population by Iliescu et al. (2015). Each scale consists of 10 items, five items are scored positive, and five items are scored negative. Examples of the items include: “I feel comfortable around people,” for extroversion, “I am very attentive to details,” for conscientiousness, and “I often feel sad,” for neuroticism. All items were scored on a five-point scale ranging from 0 (strongly disagree) to 4 (strongly agree). For the present sample, scales had adequate fit measures in terms of construct validity, considering the small sample size [χ^2^ = 1268.12, df = 402, *p* < 0.001, CFI = 0.77, TLI = 0.75; RMSEA = 0.08 (0.08, 0.09), SRMR = 0.08] and excellent internal consistency as reliability indicator (α = 0.87 for extroversion; α = 0.78 for conscientiousness, and α = 0.86 for neuroticism).

Technology-related teaching skills were measured with the technological pedagogical content knowledge scale (TPCK) from the TPACK questionnaire (Schmidt et al., 2009). The scale contains five items scored on a five-point scale ranging from 1 (strongly disagree) to 5 (strongly agree). An example item is “I can select technologies to use in my classroom that enhance what I teach, how I teach, and what students learn.” The scale was used in other cultural contexts as a measure of aspects related to ICT in cases of teachers (Abbitt, 2011; Chai et al., 2011; Yurdakul et al., 2012; Rienties et al., 2013). It was checked for accuracy using the standard back-translation technique (Brislin, 1970). In terms of validity, CFA indicated a very good fit of measures [χ^2^ = 21.36, df = 5, *p* < 0.001, CFI = 0.98, TLI = 0.97; RMSEA = 0.10 (0.06, 0.15), SRMR = 0.02]. Cronbach’s alpha value of the scale for the present sample reveals an excellent internal consistency (α = 0.92).

Coping strategies were measured using the COPE Inventory developed by Carver et al. (1989). The questionnaire consists of 48 items developed to measure 12 different coping strategies. The present study used the score for second-order factors as recommended by Carver et al. (1989) from the Romanian version of COPE Inventory adapted by Crasovan and Sava (2013). The coping mechanisms measured are as follows: (1) coping focus on the problem (including active approach, planning, and deletion of concurrent activities as coping strategies); (2) coping focus on emotions (with positive interpretation and growth, restraint, and acceptance as component strategies); (3) coping focus on social support (use of the social-instrumental support, the social-emotional support, and focusing on expressing emotions scales), and (4) avoidance coping (containing denial, mental, and behavioral deactivation as scales). Examples of items include: “I concentrate my efforts on doing something about it,” for active approach as a part of problem-focused coping; “I force myself to wait for the right time to do something,” for restraint as emotion-focused coping; “I ask people who have had similar experience of what they did,” for the use of the social-instrumental support as social support focused coping; or “I go to movies or watch TV, to think about it less,” for mental deactivation as an avoidant coping mechanism. The answers were measured on a Likert scale from 1 (“I usually don’t do this at all”) to 4 (“I usually do this a lot”). The COPE inventory is regarded as a standard norm for measuring coping strategies and ability to self-regulate in response to different experienced stressors (MacIntyre et al., 2020; Agha, 2021; Gurvich et al., 2021). The present study used the dispositional or trait-like version in which respondents report the extent to which they usually do the things listed when they are stressed. The CFA for construct validity verification indicated adequate fit measures [χ^2^ = 340.43, df = 48, *p* < 0.001, CFI = 0.78, TLI = 0.70; RMSEA = 0.14 (0.13, 0.16), SRMR = 0.14] for the present sample, considering negative correlation between the factors themselves. Cronbach’s alpha value of the scales as reliability measure is 0.73 for emotion-focused coping, 0.79 for social support-focused coping, and 0.80 for both avoidant- and problem-focused coping.

### Data Analyses

To assess the validity of our measures and verify the possible occurrence of common method bias (Podsakoff et al., 2012), a CFA using the Lavaan package (Rosseel, 2012) in R (R Core Team, 2021) was conducted for the first and for the second hypothesis. Two measurement models: M1, a model with four factors (extroversions, conscientiousness, neuroticism, and TPACK), and M2, a single factor model for the first hypothesis (Podsakoff et al., 2012) were compared. The same algorithm was maintained for the second hypothesis where an eight-factor measurement model, M3 (extroversions, conscientiousness, neuroticism, TPACK, problem-focused coping, emotion-focused coping, social support-focused coping, avoidant coping, job-affective well-being, and burnout) and a single factor model, M4 were compared. Model fit was evaluated using a maximum likelihood estimation; also, two relative fit indices (TLI and CFI), and three absolute fit indices (the chi-square statistic; SRMR, and RMSEA) was calculated. The cut-off values for the acceptable fit are as follows: RMSEA < 0.05 and SRMR < 0.05; CFI and TLI > 0.90 (Marsh et al., 2004).

Two hierarchical linear regression analyses were implemented to test the first hypothesis, where job-related affective well-being and burnout had been introduced as dependent variables. Based on the theoretical and empirical arguments, personality traits and technology-related teaching skills were introduced as predictor variables. Age, gender, marital status, and level of teaching show no correlation with the dependent variable for the present sample. As a result, they were not introduced as predictors. The analyses were carried out using the same procedure for each of the two outcomes separately. The same algorithm was followed for both the dependent variables: in the first step, extroversion, conscientiousness, and neuroticism were introduced. After controlling the influence of personality traits, TPACK as technology-related skill was introduced. The regression analyses were performed in SPSS 23.0.

Based on the results from the first two hypotheses, a mediation analysis for each combination of IV, MV, and DV to verify the mediating role of each cognitive strategy in the associations between the personality traits and teacher well-being indicators was performed. To test the third hypothesis, the twenty-eight equations of regression that resulted were analyzed using the Lavaan package (Rosseel, 2012) in R (R Core Team, 2021). Hierarchical regression analysis allows for the estimation of the unique contribution of an independent variable, above and beyond what is explained by other variables (Field, 2009, p. 212). Mediation analysis allows the researchers to distinguish and estimate the direct, indirect, and total effects of an independent variable on a dependent variable (Kenny, 2008). For the present research, Baron and Kenny’s (1986) analysis technique was used following the conditions for establishing a mediation. The requirements for hierarchical linear regression (Field, 2009, p. 212) and two of the three requirements for “measurement-of-mediation” design (Cook et al., 2002) were fulfilled. Even though teachers are nested into schools and some schools might provide better access to technology and technology-related knowledge (thus implying a multilevel data structure), the author expects that the individual responses of the teachers are to be largely independent of each other. The limitation of the present study relates to the “measurement-of-mediation” method regarding the third requirement which states that no plausible alternative explanations account for the relation between the hypothesized causal and outcome variables (Cook et al., 2002). However, other variables like technology acceptance (Shamsi et al., 2021) could confound the relationship between coping strategies and job-related well-being indicators and might be an important limitation for the validity of the proposed model of mediation (Green et al., 2010; Pirlott and MacKinnon, 2016). Future research could replicate the proposed mediation model using a manipulation-of-mediation design which further strengthens the ability to infer that those coping mechanisms were the variables responsible for the process by which personality traits affected job-related affective well-being and burnout (Crano et al., 2014).

## Results

### Measurement Models

The first model (M1) had acceptable fit indices, χ^2^(734) = 2005.30, *p* < 0.001, CFI = 0.79; TLI = 0.78; RMSEA = 0.07, CI (0.07, 0.08), SRMR = 0.07. The common factor model (M2) displayed poor fit: χ^2^(740) = 4014.9, *p* < 0.001; CFI = 0.48; TLI = 0.45; RMSEA = 0.12, CI (0.121, 0.129), SRMR = 0.11. The chi-square difference test indicated that M1 fit the data better than M2, Δχ^2^(6) = 2009.6, *p* < 0.001.

### Regression Analyses

Table 2 presents the means, the standard deviations, the internal consistency, and the correlations established between the variables in the model. All predictors show significant correlations with the dependent variables, job-related affective well-being, and burnout, respectively.

**TABLE 2:** Means, standard deviations, and correlation coefficients between variables (*N* = 284).

Variables	*M*	*SD*	1.	2.	3.	4.	5.	6.	7.	8.	9.
1. Extroversion	24.38	6.00	(0.87)								
2. Conscientiousness	30.01	4.78	0.46**	(0.78)							
3. Neuroticism	11.17	6.25	−0.55**	−0.57**	(0.86)						
4. TPCK	20.53	2.92	0.28**	0.41**	−0.41**	(0.92)					
5. Problem focused coping	39.99	4.31	0.27**	0.35**	−0.35**	0.37**	(0.80)				
6. Emotion focused coping	38.36	4.17	0.23**	0.27**	−0.38**	0.33**	0.58**	(0.73)			
7. Social support focused coping	33.90	4.92	0.03	−0.22**	0.17**	–0.01	0.12*	0.18**	(0.79)		
8. Avoidant coping	24.00	5.16	−0.30**	−0.50**	0.43**	−0.27**	−0.38**	−0.12**	−0.25**	(0.79)	
9. Job-related affective WB	72.64	16.3	0.42**	0.48**	−0.53**	0.30**	0.20**	0.16**	−0.16**	−0.31**	(0.95)
10. Burnout	17.94	11.4	−0.45**	−0.55**	0.63**	−0.31**	−0.22**	−0.17**	0.19**	0.35**	−0.66 (0.91)

*p < 0.05, **p < 0.01, one single tailed. Internal consistency alphas are displayed diagonally.

For the first hypothesis which states that extroversion and conscientiousness are positively correlated with job-related affective well-being, neuroticism is negatively correlated with job-related affective well-being (H1a); also, extroversion and conscientiousness are negatively correlated with burnout, while neuroticism is positively correlated with burnout (H1b), and all correlations are in the expected direction. Extroversion and conscientiousness correlated positively and significantly with job-related affective well-being (*r* = 0.42, r = 0.48, *p* < 0.01) and negatively and significantly with burnout (*r* = −0.45, r = −0.55, *p* < 0.01). Neuroticism correlated negatively and significantly with job-related affective well-being (r = −0.53, *p* < 0.01) and positively with burnout (*r* = 0.63, *p* < 0.01).

For the second hypothesis which assumes that TPCK is positively correlated with job-related affective well-being (H2a) and negatively correlated with burnout (H2b), correlation is positive and significant with job-related affective well-being (*r* = 0.30, *p* < 0.01) and negative with burnout (*r* = −0.31, *p* < 0.01).

Table 3 shows the results of the hierarchical regression with the two dependent variables. Concerning job-related affective well-being, in the first step, personality traits contributed to the variance of the dependent variable in the proportion of 12% [*R* = 0.58; *F*(3,280) = 48.94, *p* < 0.01]. Each of the personality traits is a significant predictor of job-related affective well-being: conscientiousness (β = 0.22, *p* < 0.01), neuroticism (β = −0.032, *p* < 0.01), and extroversion (β = 0.13, *p* < 0.05). In the second step, adding TPACK leads to explaining the variance of the job-related affective well-being in the proportion of 3% [*R* = 0.59; *F*(4,279) = 31.14, *p* < 0.01] and accounted for an additional 3% of the variance compared to the first model (β = 0.18, *p* < 0.05). In the second equation, personality traits were also a significant predictor (β = 0.23, *p* < 0.01 for conscientiousness, β = −0.30, *p* < 0.01 for neuroticism, β = 0.13, *p* < 0.01 for extroversion). Concerning burnout, in the first step, personality traits contributed with the variance of the dependent variable in the proportion of 11% [*R* = 0.67; *F*(3,280) = 18.39, *p* < 0.01]. Each of the personality traits is a significant predictor of burnout: conscientiousness (β = −0.25, *p* < 0.01), neuroticism (β = 0.42, *p* < 0.01), and extroversion (β = −0.10, *p* < 0.05). Regarding technology-related teaching skills, despite a strong correlation between TPCK and burnout (r = −0.31, *p* < 0.01) the adding of it in the second step did not improve the model and no significant correlation was found between TPCK and burnout (H1b). The statistical support was totally received in case of job-related well-being, and partially for burnout in the case of the first hypothesis. The effect size of the β coefficient is provided in Table 3 and was calculated following Acock’s (2014) suggestion.

**TABLE 3:** Hierarchical multiple regression analyses (*N* = 284).

Variables	Job-related affective well-being			Burnout		
	*R*/*R*^2^/Δ*R*^2^	β	Eff. size	*R*/*R*^2^/Δ*R*^2^	β	Eff. size
*Step 1*	0.58/0.34/0.34**		Large	0.67/0.45/0.45**		Large
Conscientiousness		0.22**	Moderate		−0.25**	Moderate
Neuroticism		−0.32**	Moderate		0.43**	Moderate
Extroversion		0.13**	Weak		−0.10*	Weak
*Step 2*	0.59/0.35/0.01*			0.67/0.45/0.44		
Conscientiousness		0.23**	Moderate		−0.25**	Moderate
Neuroticism		−0.30**	Moderate		0.42**	Moderate
Extroversion		0.13*	Weak		−0.10**	Weak
TPCK		0.18*	Weak		–0.00	

*p < 0.05; **p < 0.01.

### Mediating Analysis

For the third hypothesis which states that problem-focused coping (H3a), emotion-focused coping (H3b), social support-focused coping (H3c), and avoidant coping (H3d) mediate the relationship between personality traits and teacher well-being indicators, all the correlations between predictors and mediators was analyzed. Except extroversion on social support-focused coping, all three personality traits are significantly related to coping strategies, and each personality factor (extroversion, conscientiousness, and neuroticism) has a different effect on coping strategies (extroversion on avoidant coping: *B* = −0.26, *p* < 0.001; extroversion on social support-focused coping: *B* = 0.02, *p* > 0.05; extroversion on problem-focused coping: *B* = 0.20, *p* < 0.001; extroversion on emotion-focused coping: *B* = −0.25, *p* < 0.001; conscientiousness on avoidant coping: *B* = −0.54, *p* < 0.001; conscientiousness on social support-focused coping: *B* = −0.23, *p* < 0.001; conscientiousness on problem-focused coping: *B* = 0.32, *p* < 0.001; conscientiousness on emotion-focused coping: *B* = 0.31, *p* < 0.001; neuroticism on avoidant coping: *B* = 0.35, *p* < 0.001; neuroticism on social support-focused coping: *B* = 0.13, *p* < 0.001; neuroticism on problem-focused coping: *B* = −0.24, *p* < 0.001, and neuroticism on emotion-focused coping: *B* = −0.25, *p* < 0.001). Further, of the four coping strategies analyzed, only avoidant coping had a significant total or partial mediating effect in relationship between the independent and dependent variables which were proposed. No mediating effect was found for problem-focused coping (H3a), emotion-focused coping (H3b), and social support focused coping (H3c) in the relationship between personality traits and teachers’ well-being indicators Since the additional effect of TPACK over personality traits was sustained by the results of the second hypothesis, the mediating role of the coping strategies in relationship between TPCK and the job-related affective well-being was analyzed. Except for the relationship between TPCK and job-related affective well-being through problem-focused coping, all predictors had a significant direct effect on the well-being indicators in the presence of coping strategies as mediators. Thus, when avoidant coping was included as a mediator, a direct significant direct effect was found in the conscientiousness and neuroticism on job-related affective well-being (*B* = 1.47, *p* < 0.001, respectively, *B* = −1.27, *p* < 0.001) but no significant indirect effect was found through avoidant coping (H3d). A direct positive significant direct effect was found in extroversion (*B* = 0.98, *p* < 0.001) and TPCK on job-related affective well-being (*B* = 1.29, *p* < 0.001) and a significant indirect effect was found through avoidant coping (*B* = 0.16, *p* < 0.001, respectively, *B* = 0.38, *p* < 0.001) which sustained the existence of a full mediation. Regarding burnout, a direct negative significant effect was found in the conscientiousness and extroversion on burnout (*B* = −1.18, *p* < 0.001, respectively, *B* = −0.73, *p* < 0.001) and a direct positive significant effect of neuroticism was found on burnout (*B* = 1.06, *p* < 0.001). Avoidant coping partially mediates the relationship between personality trait and burnout, providing the following coefficients: *B* = −0.13, *p* < 0.05 for indirect effect of conscientiousness on burnout, *B* = 0.08, *p* < 0.05 for indirect effect of neuroticism on burnout, and *B* = −0.13, *p* < 0.001 for the indirect effect in the extroversion on burnout. The effect size of the β coefficient was calculated following Acock’s (2014) indication. Table 4 depicts unstandardized coefficients for each equation of regression and the effect size interpretation.

**TABLE 4:** Direct, indirect, and total effects of personality dimensions and TPCK on job-related well-being and burnout through coping styles.

Equation of regression	Predictor	Path	Estimate (*B*)	SE	β
1.	*Avoidant coping* on: *Conscientiousness*	a_1_	−0.54**	0.05	–0.05
	*Job-related affective well-being* on: *Avoidant coping*	b_1_	–0.31	0.18	–0.09
	Direct effect of *Conscientiousness* on: *Job-related affective well-being*	c_1_′	1.47**	0.20	0.43
	Indirect effects through *Avoidant coping Job-related affective well-being* on: *Conscientiousness*	a_1_ + b_1_	0.16	0.10	0.04
2.	*Avoidant coping* on: *Neuroticism*	a_2_	0.35**	0.04	0.43
	*Job-related affective well-being* on: *Avoidant coping*	b_2_	–0.32	0.17	–0.10
	Direct effect of *Neuroticism* on: *Job-related affective well-being*	c_2_′	−1.27**	0.14	–0.49
	Indirect effects through *Avoidant coping Job-related affective well-being* on: *Neuroticism*	a_2_ + b_2_	–0.11	0.06	–0.04
3.	*Avoidant coping* on: *Extroversion*	a_3_	−0.26**	0.04	–0.30
	*Job-related affective well-being* on: *Avoidant coping*	b_3_	−0.64**	0.17	–0.20
	Direct effect of *Extroversion* on: *Job-related affective well-being*	c_3_′	0.98**	0.14	0.36
	Indirect effects through *Avoidant coping Job-related affective well-being* on: *Extroversion*	a_3_ + b_3_	0.16**	0.05	0.06
4.	*Avoidant coping* on: *TPCK*	a_4_	−0.48**	0.10	–0.27
	*Job-related affective well-being* on: *Avoidant coping*	b_4_	−0.79**	0.18	–0.25
	Direct effect of *TPCK* on: *Job-related affective well-being*	c_4_′	1.29**	0.31	0.23
	Indirect effects through *Avoidant coping Job-related affective well-being* on: *TPCK*	a_4_ + b_4_	0.38**	0.11	0.06
5.	*Social support focused coping* on: *Conscientiousness*	a_5_	−0.23**	0.05	–0.22
	*Job-related affective well-being* on: *Social support focused coping*	b_5_	–0.19	0.17	–0.05
	Direct effect of *Conscientiousness* on: *Job-related affective well-being*	c_5_′	1.59**	0.18	0.47
	Indirect effects through *Social support focused coping Job-related affective well-being* on: *Conscientiousness*	a_5_ + b_5_	0.04	0.04	0.01
6.	*Social support focused coping* on: *Neuroticism*	a_6_	0.13**	0.04	0.17
	*Job-related affective well-being* on: *Social support focused coping*	b_6_	–0.23	0.16	–0.07
	Direct effect of *Neuroticism* on: *Job-related affective well-being*	c_6_′	−1.36**	0.13	–0.52
	Indirect effects through *Social support focused coping Job-related affective well-being* on: *Neuroticism*	a_6_ + b_6_	–0.03	0.02	–0.01
7.	*Social support focused coping* on: *Extroversion*	a_7_	0.02	0.04	0.03
	*Job-related affective well-being* on: *Social support focused coping*	b_7_	−0.58**	0.17	–0.17
	Direct effect of *Extroversion* on: *Job-related affective well-being*	c_7_′	1.17**	0.14	0.43
	Indirect effects through *Social support focused coping Job-related affective well-being* on: *Extroversion*	a_7_ + b_7_	–0.01	0.02	–0.00
8.	*Social support focused coping* on: *TPCK*	a_8_	–0.02	0.10	–0.01
	*Job-related affective well-being* on: *Social support focused coping*	b_8_	−0.52**	0.18	–0.15
	Direct effect of *TPCK* on: *Job-related affective well-being*	c_8_′	1.66**	0.31	0.29
	Indirect effects through *Social support focused coping Job-related affective well-being* on: *TPCK*	a_8_ + b_8_	0.01	0.05	0.00
9.	*Problem focused coping* on: *Conscientiousness*	a_9_	0.32**	0.05	0.35
	*Job-related affective well-being* on: *Problem focused coping*	b_9_	0.14	0.20	0.04
	Direct effect of *Conscientiousness* on: *Job-related affective well-being*	c_9_′	1.59**	0.18	0.46
	Indirect effects through *Problem focused coping Job-related affective well-being* on: *Conscientiousness*	a_9_ + b_9_	0.04	0.06	0.01
10.	*Problem focused coping* on: *Neuroticism*	a_10_	−0.24**	0.03	–0.35
	*Job-related affective well-being* on: *Problem focused coping*	b_10_	0.07	0.19	0.01
	Direct effect of *Neuroticism* on: *Job-related affective well-being*	c_10_′	−1.37**	0.13	–0.52
	Indirect effects through *Problem focused coping Job-related affective well-being* on: *Neuroticism*	a_10_ + b_10_	–0.01	0.04	–0.00
11.	*Problem focused coping* on: *Extroversion*	a_11_	0.20**	0.04	0.27
	*Job-related affective well-being* on: *Problem focused coping*	b_11_	0.34	0.20	0.09
	Direct effect of *Extroversion* on: *Job-related affective well-being*	c_11_′	1.08**	0.15	0.39
	Indirect effects through *Problem focused coping Job-related affective well-being* on: *Extroversion*	a_11_ + b_11_	0.07	0.04	0.02
12.	*Problem focused coping* on: *TPCK*	a_12_	0.56**	0.08	0.37
	*Job-related affective well-being* on: *Problem focused coping*	b_12_	0.39	0.22	0.10
	Direct effect of *TPCK* on: *Job-related affective well-being*	c_12_′	1.45	0.33	0.26
	Indirect effects through *Problem focused coping Job-related affective well-being* on: *TPCK*	a_12_ + b_12_	0.22	0.12	0.04
13.	*Emotion focused coping* on: *Conscientiousness*	a_13_	0.24**	0.04	0.27
	*Job-related affective well-being* on: *Emotion focused coping*	b_13_	0.12	0.21	0.03
	Direct effect of *Conscientiousness* on: *Job-related affective well-being*	c_13_′	1.61**	0.18	0.47
	Indirect effects through *Emotion focused coping Job-related affective well-being* on: *Conscientiousness*	a_13_ + b_13_	0.03	0.05	0.00
14.	*Emotion focused coping* on: *Neuroticism*	a_14_	−0.25**	0.03	–0.38
	*Job-related affective well-being* on: *Emotion focused coping*	b_14_	–0.18	0.21	–0.04
	Direct effect of *Neuroticism* on: *Job-related affective well-being*	c_14_′	−1.44**	0.14	–0.55
	Indirect effects through *Emotion focused coping Job-related affective well-being* on: *Neuroticism*	a_14_ + b_14_	0.04	0.05	0.01
15.	*Emotion focused coping* on: *Extroversion*	a_15_	0.16**	0.04	0.23
	*Job-related affective well-being* on: *Emotion focused coping*	b_15_	0.26	0.21	0.06
	Direct effect of *Extroversion* on: *Job-related affective well-being*	c_15_′	1.11**	0.14	0.40
	Indirect effects through *Emotion focused coping Job-related affective well-being* on: *Extroversion*	a_15_ + b_15_	0.04	0.03	0.01
16.	*Emotion focused coping* on: *TPCK*	a_16_	0.47**	0.08	0.33
	*Job-related affective well-being* on: *Emotion focused coping*	b_16_	0.28	0.23	0.07
	Direct effect of *TPCK* on: *Job-related affective well-being*	c_16_′	1.54**	0.33	0.27
	Indirect effects through *Emotion focused coping Job-related affective well-being* on: *TPCK*	a_16_ + b_16_	0.13	0.11	0.02
17.	*Avoidant coping* on: *Conscientiousness*	a_17_	−0.54**	0.05	–0.50
	*Burnout* on: *Avoidant coping*	b_17_	0.24*	0.12	0.10
	Direct effect of C*onscientiousness* on: *Burnout*	c_17_′	−1.18**	0.13	–0.49
	Indirect effects through *Avoidant* coping *Burnout* on: *Conscientiousness*	a_17_ + b_17_	−0.13*	0.06	–0.05
18.	*Avoidant coping* on: *Neuroticism*	a_18_	0.35**	0.04	0.43
	*Burnout* on: *Avoidant coping*	b_18_	0.22*	0.11	0.10
	Direct effect of *Neuroticism* on: *Burnout*	c_18_′	1.06**	0.09	0.58
	Indirect effects through *Avoidant* coping *Burnout* on: *Neuroticism*	a_18_ + b_18_	0.08*	0.04	0.04
19.	*Avoidant coping* on: *Extroversion*	a_19_	−0.26**	0.04	–0.30
	*Burnout* on: *Avoidant coping*	b_19_	0.53**	0.11	0.24
	Direct effect of *Extroversion* on: *Burnout*	c_19_′	−0.73**	0.10	–0.38
	Indirect effects through *Avoidant* coping *Burnout* on: *Extroversion*	a_19_ + b_19_	−0.13**	0.04	–0.07
20.	*Social support focused coping* on: *Conscientiousness*	a_20_	−0.23**	0.05	–0.22
	*Burnout* on: *Social support focused coping*	b_20_	0.18	0.11	0.08
	Direct effect of *Conscientiousness* on: *Burnout*	c_20_′	−1.27**	0.12	–0.53
	Indirect effects through *Social support focused coping Burnout* on: *Conscientiousness*	a_20_ + b_20_	–0.04	0.02	–0.01
21.	*Social support focused coping* on: *Neuroticism*	a_21_	0.13**	0.04	0.17
	*Burnout* on: *Social support focused*	b_21_	0.21**	0.10	0.09
	Direct effect of *Neuroticism* on: *Burnout*	c_21_′	1.12**	0.08	0.61
	Indirect effects through *Social support focused coping Burnout* on: *Neuroticism*	a_21_ + b_21_	0.02	0.01	0.01
22.	*Social support focused* on: *Extroversion*	a_22_	0.02	0.04	0.03
	*Burnout* on: *Social support focused coping*	b_22_	0.5**	0.11	0.21
	Direct effect of *Extroversion* on: *Burnout*	c_22_′	−0.88**	0.09	–0.46
	Indirect effects through *Social support focused Burnout* on: *Extroversion*	a_22_ + b_22_	0.01	0.02	0.00
23.	*Problem focused coping* on: *Conscientiousness*	a_23_	0.32**	0.05	0.35
	*Burnout* on: *Problem focused coping*	b_23_	–0.08	0.13	–0.03
	Direct effect of C*onscientiousness* on: *Burnout*	c_23_′	−1.29**	0.12	–0.54
	Indirect effects through *Problem focused coping Burnout* on: *Conscientiousness*	a_23_ + b_23_	–0.02	0.04	–0.01
24.	*Problem focused coping* on: *Neuroticism*	a_24_	−0.24**	0.03	–0.35
	*Burnout* on: *Problem focused coping*	b_24_	–0.00	0.12	–0.00
	Direct effect of *Neuroticism* on: *Burnout*	c_24_′	1.15**	0.09	0.62
	Indirect effects through *Problem focused coping Burnout* on: *Neuroticism*	a_24_ + b_24_	0.00	0.03	0.00
25.	*Problem focused coping* on: *Extroversion*	a_25_	0.20**	0.04	0.27
	*Burnout* on: *Problem focused coping*	b_25_	–0.26	0.14	–0.10
	Direct effect of *Extroversion* on: *Burnout*	c_25_′	−0.81**	0.10	–0.43
	Indirect effects through *Problem focused coping Burnout* on: *Extroversion*	a_25_ + b_25_	–0.05	0.03	–0.02
26.	*Emotion focused coping* on: *Conscientiousness*	a_26_	0.24**	0.04	0.27
	*Burnout* on: *Emotion focused coping*	b_26_	–0.05	0.14	–0.02
	Direct effect of *Conscientiousness* on: *Burnout*	c_26_′	−1.30**	0.12	–0.54
	Indirect effects through *Emotion focused coping Burnout* on: *Conscientiousness*	a_26_ + b_26_	–0.01	0.03	–0.00
27.	*Emotion focused coping* on: *Neuroticism*	a_27_	−0.25**	0.03	–0.38
	*Burnout* on: *Emotion focused coping*	b_27_	0.21	0.13	0.08
	Direct effect of *Neuroticism* on: *Burnout*	c_27_′	1.2**	0.09	0.66
	Indirect effects through *Emotion focused coping Burnout* on: *Neuroticism*	a_27_ + b_27_	–0.05	0.03	–0.03
28.	*Emotion focused coping* on: *Extroversion*	a_28_	0.16**	0.04	0.23
	*Burnout* on: *Emotion focused coping*	b_28_	–0.19	0.14	–0.06
	Direct effect of *Extroversion* on: *Burnout*	c_28_′	−0.84**	0.10	–0.44
	Indirect effects through *Emotion focused coping Burnout* on: *Extroversion*	a_28_ + b_28_	–0.03	0.02	–0.01

*p < 0.05; **p < 0.01.

## Discussion

Decisions on how to promote teachers’ job-related well-being while working on-line at home need to be based on the best available evidence. This study aimed to identify a model of prediction for burnout and job-related affective well-being during online teaching. Based on the TPACK model, the additional value of TPCK as technology-related teaching skills in explaining the above-mentioned indicators of well-being in an online teaching setting, over and above personality traits were studied. In addition, following the adaptation mechanism initially proposed by the transactional model of stress, mediating role of coping strategies between personality traits and well-being indicators during online teaching in the COVID pandemic was also studied.

The analysis of the data in the study indicated that the first model which includes personality traits together has a stronger prediction power on the job-related affective well-being and burnout because the effect size is large in both the cases. However, although TPCK is reported as statistically significant as a predictor for job-related affective well-being, over and above the personality traits, the effect size is weak. Based on the findings obtained in the current sample, the results cannot be considered educationally significant. Future replication studies might be conducted on larger samples or may include other parameters related to technology. In line with a previous study (DeNeve and Cooper, 1998; Anglim et al., 2020; Zager Kocjan et al., 2021), personality traits predict teachers’ well-being indicators as it was expected. In case of job-related affective well-being, the strongest predictor is neuroticism followed by conscientiousness and extroversion. By adding TPACK, the prediction model becomes stronger although the effect size is small for this sample. Regarding burnout, the strongest predictor was neuroticism followed by conscientiousness and extroversion. Neuroticism negatively predicted job-affective well-being and positively burnout, while conscientiousness and extroversion are positive predictors for job-affective well-being and a negative predictor for burnout. The review of the literature indicated that neuroticism and extroversion are nearly identical to two elements of subjective well-being, negative and positive affect, respectively; the neurotic individuals tend to be anxious or depressed, whereas the extroverts tend to be sociable, optimistic, and energetic (Steel et al., 2008). Extroversion is an orientation of one’s interests and energies toward the outer world of people and things rather than the inner world of subjective experience (VandenBos and American Psychological Association, 2007). Thus, a low prediction power of extroversion on teachers’ well-being indicators may be explained by the fact that social connectedness, a variable affected by the pandemic conditions is a significant mediator explaining the relationship between extroversion and perceived well-being (De Raad, 2000; Lee et al., 2008). Moreover, individuals with high extroversion reported higher levels of distress because they may not be as effective in controlling their environment once the social aspect is removed (Abbott et al., 2008). Also, the extroverted personalities were associated with telecommunication burnout, whereas introverts were found to face stress resulting from telecommunication more easily (Meymandpour and Bagheri, 2017). Neuroticism, on the other side, is characterized by a chronic level of emotional instability and proneness to psychological distress (VandenBos and American Psychological Association, 2007). Like Steel et al. (2008) the current findings showed that neuroticism was the most consistent correlate of subjective well-being followed by extroversion and then conscientiousness. Conscientiousness represents the tendency to be organized, responsible, and hardworking (VandenBos and American Psychological Association, 2007). Thus, people with higher levels of conscientiousness follow COVID-19 preventive measures rigorously. Consequently, this quality may further enhance their coping resources to prevent COVID-19 while minimizing their perceived threat of COVID-19, and resulting in lower stress (Vollrath, 2001). Thus, I can deduce that conscientious teachers are lower in burnout and higher in job-related affective well-being since their consistency and strictness trigger efficient control of stress.

The results outlined that TPCK predicted higher subjective well-being over and above personality traits in online teaching settings. Although the influence of the digital technologies knowledge in teacher well-being has been scarcely researched (Passey, 2021), some previous results argue that the TPACK model influenced the existence of technostress in the teachers (Joo et al., 2016). Thus, the findings are in line with a recent study that highlights that the use of educational technology in the classroom is associated with higher levels of anxiety or stress (Fernández-Batanero et al., 2021). One can only assume that teachers with high scores of TPCK might be more confident and less stressed in online teaching settings resulting in enchanted job-related affective well-being and low burnout. However, although the magnitude of effect is low for the current sample, this study expands the research on the implications of technology-related teaching skills for teacher well-being in an educational setting advocating for their role in increasing job-related affective well-being because they are malleable and can be improved. Future studies could analyze in more depth on different samples regarding the role of TPCK and other technology-related teaching skills for job-related well-being.

Previous research has focused on coping strategies and how they can alleviate stress levels and promote a higher quality of life at work (Acker, 2018; Cancio et al., 2018). Coping strategies can represent a valuable resource for teachers dealing with stressors, and research has indicated that the nature and context of stress influence the relation between personality and coping because coping is tailored to match the demands of specific situations (Lee-Baggley et al., 2005). The present study paid attention to the potential impact in the well-being domain of response-coping strategies to a specific stressor which is the request to teachers to adapt and to be efficient in online teaching without previous training. Personality should also be strongly linked to dispositional coping because personality influences the type of events experienced, which in turn influences typical coping (Bouchard et al., 2004). The present results have shown that all three personality traits are significantly related to coping strategies, and each personality factor (extroversion, conscientiousness, and neuroticism) has a different effect on coping strategies. Therefore, personality traits may influence the effectiveness of coping strategies, with strategies that are beneficial for some individuals being less effective, or even harmful, for those with different personality traits (Bolger and Zuckerman, 1995; Hudek-Knežević et al., 2005). Consistent partially with in the study by Carver and Connor-Smith (2010), the current findings highlight that personality and avoidant coping play both independent and interactive roles in influencing the well-being indicators. Further, results revealed that high levels of extroversion and TPCK predicted a high level of job-related affective well-being while high levels of conscientiousness predicted lower burnout. On the other hand, neuroticism predicted increased burnout. However, appealing to avoidant coping, the dynamic of relationship between personality traits on the two well-being indicators is changing but it has a weak magnitude of effect on all the three indirect effects of personality traits (extroversion, neuroticism, and conscientiousness) on burnout and for extraversion and TPCK effects on job-related affective well-being through avoidant coping. The coping literature conceptualizes avoidance coping as a maladaptive (or unhealthy) coping strategy because it often exacerbates stress without helping a person deal with the things that cause the stress (e.g., Ingledew et al., 1997; Dijkstra and Homan, 2016). On the contrary, the work recovery literature considers psychological detachment as an adaptive strategy that can help individuals deal with stress (Sonnentag et al., 2008). Therefore, studies that examined avoidance coping provided mixed findings. Andreassi (2011) reported that avoidance coping has detrimental effects while another research has found it to be beneficial (Hecht and McCarthy, 2010; Rantanen et al., 2011). Since the current research provides just one score for avoidance coping measured through three avoidance coping strategies as second-order factors (denial, mental deactivation, and behavioral deactivation), it can be assumed that avoidance coping contains two underlying components (Cheng and McCarthy, 2013). In our case, one component deals with stressors by cognitively or behaviorally distancing from the situation (cognitive and behavioral avoidance) and the other one concerns a distorted view of reality that involves a kind of fanciful thinking (escape avoidance; Folkman and Lazarus, 1985) named denial in the present research. Cognitive avoidance is posited to be beneficial as it serves to replenish depleted resources that can be redirected toward various tasks (Hobfoll, 1989) since it reflects a mental distancing from a stressor (Cheng and McCarthy, 2013). Thus, in case of high neuroticism, avoidant coping (especially throughout cognitive deactivation) may buffer against detrimental effects of adverse situations and, at the same time, improve the subjective well-being of individuals by decreasing burnout. Although the magnitude of effect is weak for the current sample, the result is consistent with the previous study which emphasizes that avoidance has predicted increased negative affect for low neurotic individuals, but not for high neurotic individuals (Bolger and Zuckerman, 1995) because neuroticism involves intense emotional and physical responses to stress linked to attempts to minimize unpleasant arousal through disengagement strategies, such as avoidance (Connor-Smith and Flachsbart, 2007). Specifically, teachers who are highly stress-reactive may disengage to decrease their own unpleasant arousal. Further, since conscientiousness involves the tendency to plan, reducing the number of stressors experienced, this personality trait may be negatively related to dispositional disengagement (Connor-Smith and Flachsbart, 2007). On the other hand, research findings indicate that individuals higher on extroversion are more prone to engage in less avoidance (McCrae and Costa, 1986). The present results support these statements because in the case of teachers who are extroverts, conscientiousness, and with a high score for TPCK, adopting avoidant coping leads to a lower job-related affective well-being and increased level of burnout.

### Implication for Teachers

The results of the current study might be of great interest to teachers because they emphasize two important ideas: the role of TPCK for teachers in the job-related affective well-being and the beneficial effect of avoidant copping for professionals with high levels of neuroticism in on-line teaching settings. Following the study of Revilla Muñoz et al. (2017) which places technology training as the best option for reducing technostress, some recommendations for teachers could include suggestions to keep an open attitude toward technological innovation and to follow tutorials for improving their technological knowledge especially if school organizations do not provide free training. The present study also emphasizes that avoidant coping strategies are effective when teachers have high levels of neuroticism. Therefore, one recommendation for teachers who know themselves as being tense and prone to negative emotions (such as anxiety, depression, or anger) is to increase the frequency of breaks between online lectures while practicing mindfulness as mental and behavioral disengagement-coping strategies. A mindfulness approach combined with releasing eye strain has proven to provide an adaptive response to stressors (Riley and Park, 2015).

The present study is not without limitations. The first limitation is that the study focused on broad categories of coping rather than on specific coping strategies, and on personality factors rather than on specific personality facets. Consequently, it is possible that some specific coping strategies could be used to deal with online teaching demands, but the present study cannot reveal them since the global dimension of a coping style has been measured. Moreover, correlations between the facets of the personality factors and coping strategies could not be analyzed. The second limitation is related to the fact that the validity of responses could have been affected due to social desirability since the present study has collected data using a set of self-report measures. In addition, demands regarding teaching levels are different and could represent another source of stress next to the necessity to adapt to online teaching but the current sample was heterogenous and relatively small for each category. Consequently, more control of hidden variables is necessary. Yet, regarding theoretical and practical implications, the present study outlines the independent and interactive roles of personality traits, technology-related teaching skills, and coping strategies in influencing teacher well-being indicators in online teaching settings during the COVID-19 pandemic. Based on psychological approaches, the struggle for digital well-being in our COVID and post-COVID world needs to enlist more strategies to improve well-being in online teaching settings, and the present study provides a deeper insight into the interplay of personality and coping. Thus, the current results could aid in the design of the more effective intervention and prevention programs fitted to the unique personality profile of individuals.

## Conclusion

Online teaching in COVID-19 pandemic brought consequences for teachers’ job-related well-being. The present study emphasizes that TPACK is a personal resource which can be enhanced and used to predict an increased level of job-related affective well-being. Moreover, avoidant-coping strategies buffer against burnout in cases of teachers with high neuroticism but an opposite effect was obtained in case of extrovert teachers. Results argue for the value of examining individual differences in variables in research on occupational stress related to the online teaching setting.

## Data Availability Statement

The raw data supporting the conclusions of this article will be made available by the authors, without undue reservation.

## Ethics Statement

The studies involving human participants were reviewed and approved by Ethics Committee for Research, Faculty of Socio-Humanistic Sciences, University of Oradea. The patients/participants provided their written informed consent to participate in this study.

## Author Contributions

The author confirms being the sole contributor of this work and has approved it for publication.

## Conflict of Interest

The author declares that the research was conducted in the absence of any commercial or financial relationships that could be construed as a potential conflict of interest.

## Publisher’s Note

All claims expressed in this article are solely those of the authors and do not necessarily represent those of their affiliated organizations, or those of the publisher, the editors and the reviewers. Any product that may be evaluated in this article, or claim that may be made by its manufacturer, is not guaranteed or endorsed by the publisher.
